# Moisture adsorption isotherms and quality of seeds stored in conventional packaging materials and hermetic Super Bag

**DOI:** 10.1371/journal.pone.0207569

**Published:** 2019-02-15

**Authors:** Muhammad Amir Bakhtavar, Irfan Afzal, Shahzad Maqsood Ahmed Basra

**Affiliations:** Seed Physiology Lab, Department of Agronomy, University of Agriculture Faisalabad, Punjab, Pakistan; Nigde Omer Halisdemir University, TURKEY

## Abstract

Seed moisture content (SMC) is an important attribute to seed quality. Maintaining seed dryness throughout supply chain (The Dry Chain) prevents seed germination and quality losses. Ambient relative humidity (RH) and temperature affect seed moisture and thereof seed moisture isotherm. Present study was conducted to compare the moisture adsorption isotherms of wheat, maize, cotton and quinoa seeds packed in hermetic Super Bag and traditional packaging materials including paper, polypropylene (PP), jute and cloth bags. Seeds were incubated at 60, 70, 80 and 90% static RH. Nearly straight line moisture isotherms for all crop seeds were obtained in Super Bag. Seed moisture contents increased in traditional packaging materials with increasing RH. At higher level of RH, moisture contents increased slightly (1–2%) in Super Bag, whereas this increase was much higher in traditional packaging materials (≈9% higher than original SMC at 90% RH). In second study, seeds were dried to 8 and 14% initial seed moisture contents using zeolite drying beads and were stored in hermetic and conventional packaging materials for a period of 18 months. For all crop seeds, germination was severely affected in all packaging materials both at 8 and 14% initial SMC except storage in Super Bag at 8% SMC. Wheat seed stored in Super Bag at 8% SMC almost maintained initial germination while germination of cotton, maize and quinoa seeds declined 7%, 14% and 30% respectively in Super Bag at 8% SMC. Seed storage in Super Bag can help to prevent the significant increase in seed moisture at higher RH as is evident from moisture isotherm study, thus helps to preserve quality of maize, wheat, cotton and quinoa seeds by maintaining The Dry Chain throughout the storage period.

## Introduction

Seed moisture is a critical factor influencing seed quality, as seed shelf life is highly dependent upon its moisture contents. Knowledge of best seed moisture content for seed storage both increases shelf life and reduces contamination by storage fungi [1]. Knowing seed moisture even in the field of seed testing is essential to avoid the imbibitional injury during germination process [2] and it is essential parameter in standardizing results of vigor test such as conductivity test and accelerated aging test [3]. Bewley et al. [4] emphasized that seed quality is at greatest risk at high moisture content during storage. Rate of seed reparation increased at high seed moisture contents and heat generated by the respiring seeds is enough to kill the seeds [5]. Storage of maize at high moisture contents (15%) resulted into low germination percentage, dry matter losses (up to 35%) along with fungal growth [6].

Seed moisture isotherm describe the equilibrium relationship between prevailing RH and SMC at a given temperature [7]. Seed moisture adsorption isotherms are an important tool to predict the potential changes occurring in the biological material’s stability [8]. Seed is a hygroscopic entity and absorbs moisture from the surrounding, so any change in temperature and RH of the surrounding, affects its moisture isotherm, moisture contents and ultimately its quality [9]. Moisture isotherms of four vegetable seeds showed a difference in water activity and seed longevity over same equilibrium moisture contents [10]. Equilibrium moisture contents of safflower seeds increased with increasing water activity (Equilibrium relative humidity/100) at specific temperature [11]. Nature of the packaging materials has also great influence over the seed moisture contents. Different packaging materials have different water vapor transmission rates [12] thus will have difference in the moisture contents of seeds contained inside them. In most of south Asian countries including Pakistan, seeds are stored in conventional porous packaging materials and earthen bins [13]. Large pore size of jute, cloth and woven polypropylene bags provide free access to the water vapors that were readily absorbed by the seeds and ultimately elevated seed moisture contents.

Maintaining seed dryness by implementing the Dry Chain reduces storage losses of stored commodities [1], thus offers an opportunity for safe storage of seeds without significant loss of germination. The Dry Chain implies an initial drying of commodity to low moisture and maintaining this low moisture throughout the supply chain. Conventional packaging materials are porous in nature and even dried seeds can regain moisture in these packaging materials under high ambient relative humidity. Super Bags are made up of high strength polyethylene (PE) with barrier layers and mostly comes in green color. Hermetic Super Bag has very low oxygen (≤ 4 cc/m^2^/day) and water vapor transmission rate (≤5 gm^-2^day^-1^) and can be used to store seeds safely [14]. Super Bag resists any significant change in seed moisture due to gas coated barrier layers. Rice seed stored in Super Bag retained high germination and grain milling quality [15]. Hermetic Super Bags reduced the storage losses of cowpea grain in Niger as compared to woven plastic bags [16]. There is no study available on seed moisture isotherm variations within Super Bag and conventional packaging materials with changing RH and its ultimate effect on seed quality. The present study was conducted to compare moisture adsorption isotherm of different crop seeds at various levels of RH when stored in conventional porous packaging materials and hermetic Super Bags. Moreover, germination of wheat, maize, cotton and quinoa seeds stored in these packaging materials at 8 and 14% initial seed moisture contents have been compared after 18 month to check if maintaining seed dryness (The Dry Chain) by storage in Super Bag could be a strategy to preserve seed quality.

## Materials and methods

### Seed moisture isotherm study

**Fig 1:**
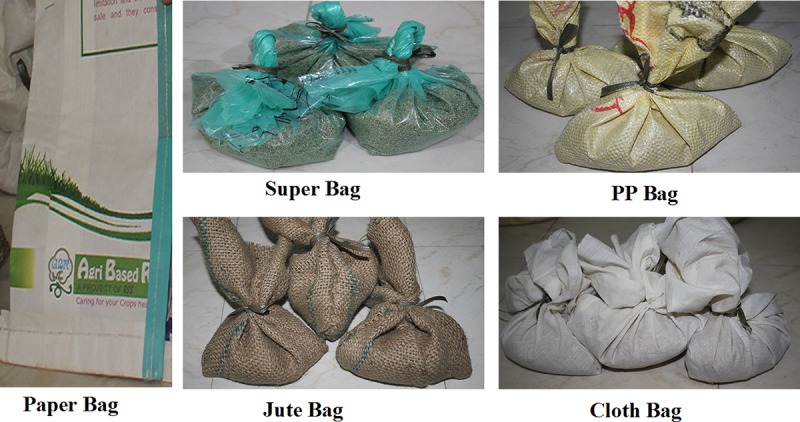
Different packaging materials used for seed moisture adsorption isotherm study.

Experiment was performed in Seed Physiology Lab, Department of Agronomy, University of Agriculture Faisalabad, Pakistan. Hermetic plastic Super Bags were provided by the GrainPro Inc. USA. Super Bag and conventional packaging materials (Fig 1) including paper bag, PP bag (woven poly propylene), cloth bag (woven cotton yarn) and jute bag (woven jute yarn) were cut into smaller size (1 kg capacity). Hermetic Super Bags were cut from top portion in a way that only bottom portion of the bag was used so as to retain its hermetic properties. Initial moisture contents of wheat and maize were 9 and 9.2% respectively, while moisture contents of quinoa and cotton seed were 7.5 and 7.65% on fresh weight basis. After packaging 150 g seeds of each crop, edges of Super Bag were twisted, folded back and tied with plastic thread. Edges of paper bags were sealed with scotch tape. All bags were incubated at four static RH levels (60, 70, 80 and 90%) maintained in airtight plastic boxes keeping a constant temperature of 25°C for the period of 20 days.

#### Maintaining static RH levels

Saturated salt solutions were used to maintain static RH levels in airtight plastic boxes. Saturated solution of FeCl_2_.4H_2_O gave 60% RH and NaCl+NaNO_3_ maintained ≈70% at 25°C. Similarly, saturated solution of NaH_2_PO_4_ maintained 80% RH while BaCl_2_.2H_2_O gave 90% RH at 25°C [17].

#### Determination of seed moisture contents

Seed moisture contents of each crop were recorded after 15 days of incubation at each of the four RH levels as seed equilibrate its moisture contents within this duration. Four replications of ≈ 5 g seeds were dried in an oven at 103°C for 17 hours [18] and dry weight of each replicate was recorded to calculate the seed moisture contents on fresh weight basis.

#### Development of moisture isotherms

Moisture isotherms were developed by plotting the data of seed moisture contents against different levels of RH (60, 70, 80 and 90%) in the form of a line graphs in Microsoft Excel.

### Seed storage study

Seed storage experiment was performed in Seed Physiology Lab, Department of Agronomy, University of Agriculture Faisalabad, Pakistan. Seed of wheat (cv. Galaxy 2013) and cotton (cv. Lalazar) was obtained from Ayub Agriculture Research Institute, Faisalabad, Pakistan. Wheat seed’s initial germination was 99.8% whereas initial germination of cottonseed was 75%. Hybrid maize (cv. 30Y87) seed was provided by Pioneer Pakistan Seeds Ltd. Sahiwal, having 99% initial germination. Quinoa (cv. UAFQ7) seed was procured form Crop Physiology Research Area, Department of Agronomy having initial 80.5% germination.

#### Seed drying

Maize and wheat seeds (5 kg per replicate) were dried to 8% seed moisture contents by mixing with zeolite seed drying beads in an airtight container. Drying beads (Rhino Research Group Thailand) are made up of aluminium silicate ceramics (Zeolite clay) material with very small, uniform pores where water molecules can be adsorbed and are used for quick drying of seeds. Amount of beads required to dry seeds was calculated using a bead calculator [1].

#### Equilibrating seed moisture contents

Maize seeds were incubated over saturated salt solution of FeCl_2_ for 20 days to maintain 14% equilibrium seed moisture contents at 25°C. To maintain 14% moisture contents, wheat seeds were incubated over saturated salt solution of NaNO_2_ at 25°C [17] for 20 days. Cotton seeds were incubated at saturated salt solution of Mg(NO_3_)_2_ and BaCl_2_ for 20 days to get 8% and 14% equilibrium seed moisture contents respectively. Similarly, quinoa seeds were incubated over saturated salt solution of CaCl_2_ and CuBr_2_ at 25°C for 20 days in airtight plastic box to get 8% and 14% seed moisture levels respectively. Seeds of each crop equilibrated to 8% and 14% seed moisture levels were packed in Super Bag, paper, PP, jute and cloth bags and were stored for 18 months in Seed Physiology Lab at ambient conditions.

#### Storage environment’s temperature and relative humidity data

Data of temperature and RH for whole storage duration (July 2015 to July 2017) were recorded with the help of Data logger (Centor Thai, Thailand) that was installed in the store house. During 2015, maximum RH was recorded in the month of November while minimum RH was recorded during October (Table 1). Maximum temperature was recorded during the month of July whereas minimum temperature was recorded in December. In 2016, maximum relative humidity was recorded in the months of January and February while minimum relative humidity was recorded during May. Maximum temperature was recorded during the month of May whereas minimum temperature was recorded in January. During 2017, maximum RH was recorded in the month of March while minimum RH was recorded during May. Maximum temperature was recorded during the month of June whereas minimum temperature was recorded in January (Table 1).

**Table 1:** Monthly maximum and minimum values of temperature and relative humidity recorded with data logger installed in seed store house.

Month	Temperature (°C)				Relative humidity (%)			
	Date	Max.	Date	Min.	Date	Max.	Date	Min.
July 2015	6 July	40.1	14 July	34.1	6 July	56.9	7 July	40.6
August 2015	31 August	39.8	24 August	31.1	24 August	65.3	9 August	43.1
Sep-2015	1 Sep.	39.9	24 Sep.	30.3	3 Sep.	60.7	11 Sep.	42.5
Oct-2015	8 Oct.	33.8	29 Oct.	24.1	26 Oct.	56.7	22 Oct.	39.8
Nov-2015	3 Nov.	25.8	30 Nov.	19.5	30 Nov.	67.1	17 Nov.	41.6
Dec-2015	1 Dec.	20.1	25 Dec	13.3	8 Dec.	61.6	15 Dec	48.4
Jan-2016	12 Jan.	17.6	26 Jan.	11.1	12 Jan.	66.3	1 Jan.	49.8
Feb-2016	29 Feb.	23.1	1 Feb	14.1	9 Feb.	66.3	18 Feb	56.3
March 2016	31 March	29	14 March	19.9	18 March	64.8	22 March	49
April 2016	30 April	36.3	11 April	25.1	4 April	65.9	30 April	37.6
May 2016	23 May	41.8	5 May	35	1 May	43.5	18 May	30.6
June 2016	7 June	41.7	23 June	36.9	22 June	44.6	6 June	33.3
July 2016	2 July	41.1	29 July	35.3	29 July	54.5	23 July	40.2
August 2016	22 August	38.7	11 August	33	11 August	59.4	18 August	45.9
Sep-2016	18 Sep.	37.5	2 Sep.	32.9	7 Sep.	50.4	27 Sep.	44.2
Oct-2016	31 Oct.	26.3	1 Oct.	36.4	31 Oct.	48.5	24 Oct.	39
Nov-2016	1 Nov.	26.5	29 Nov	20.8	22 Nov.	55.5	28 Nov.	41.5
Dec-2016	1 Dec.	21.3	27 Dec	16.5	19 Dec.	56.9	2 Dec	45.2
Jan-2017	5 Jan.	17.2	17 Jan.	13.2	25 Jan.	64.7	11 Jan.	49.6
Feb-2017	28 Feb	22.8	1 Feb.	16.1	26 Feb.	59.1	2 Feb.	53.1
March 2017	15 March	19.9	31 March	32.3	19 March	64.8	30 March	43.9
April 2017	22 April	37.4	9 April	28	14 April	52.7	30 April	27.8
May 2017	4 May	33.1	28 May	40.1	30 May	38.9	2 May	20.8
June 2017	6 June	40.9	22 June	33.8	29 June	50.5	1 June	27.2
July 2017	14 July	35	18 July	39.2	31 July	56	4 July	45.3

#### Seed germination testing

Seed sampling was done after 18 months of storage. Seed germination was tested by placing 100 seeds in sterilized and well moist blotting paper and making four similar replications to test total 400 seeds from randomly drawn seed samples. Germination test of wheat seed was conducted in a seed germinator (MIR-254, SANYO, Japan) at 20°C to provide ideal conditions [18]. For testing germination of maize, cotton and rice seed, germination temperature of the seed germinator was set to 25°C. Seedlings were evaluated according to guidelines given in ISTA Handbook for seedling evaluation [19] and percentage of normal seedlings was reported as final germination.

#### Statistical analysis

Completely Randomized Design with factorial arrangements was used for data analysis using statistical software Statistix 8.1. Seed moisture contents and packaging material were treated as factors and each experimental unit was replicated three times. Treatment means were compared using Tukey’s Test at P ≤ 0.05.

## Results

### Moisture Isotherm study

**Fig 2:**
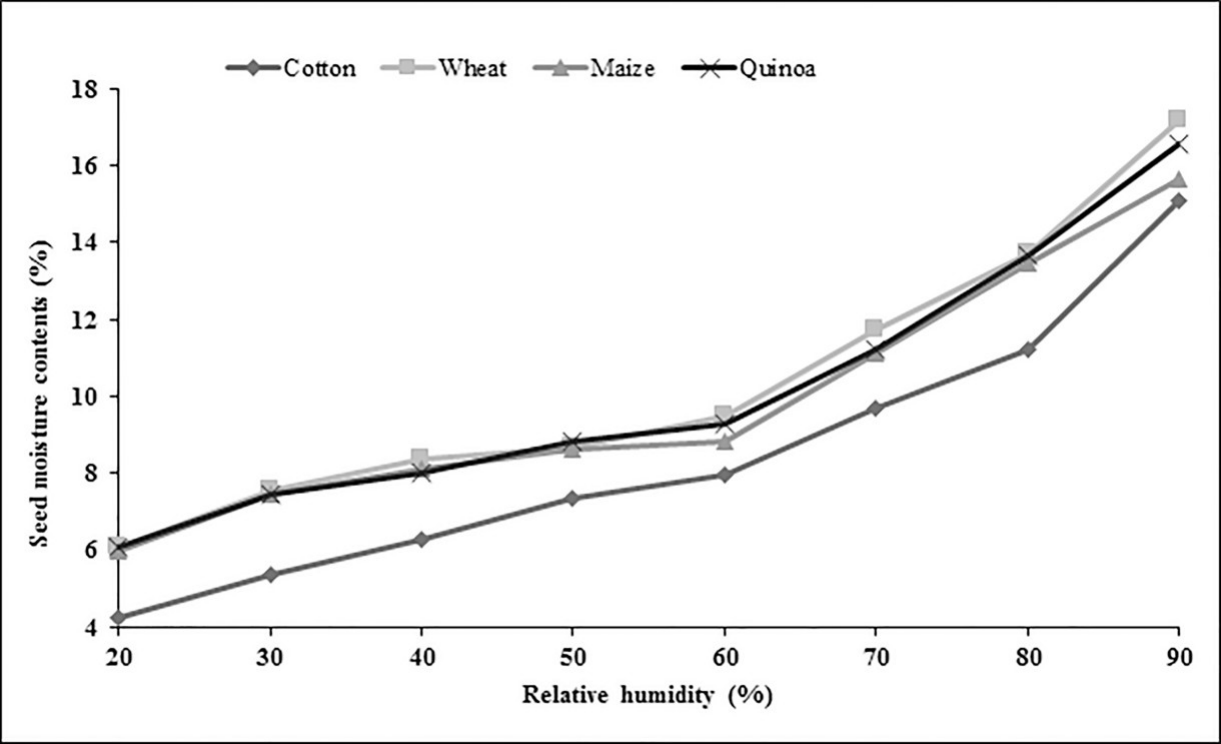
Seed moisture adsorption isotherms of cereals, psudocereals and oilseeds at 25°C.

Cottonseed having highest oil contents on percent weight basis had lowest seed moisture contents at all levels of RH (Fig 2). At 20% RH wheat seeds had highest moisture contents (6.1%) whereas cottonseeds had lowest moisture contents (4.25%). Similarly, cottonseeds had lowest moisture at 90% RH and wheat seeds had highest moisture contents at this humidity level. Maize and quinoa seed had moisture contents higher than cotton seeds with moisture contents more close to the wheat seeds at all levels of RH and slight overlapping at some points (Fig 2).

**Fig 3:**
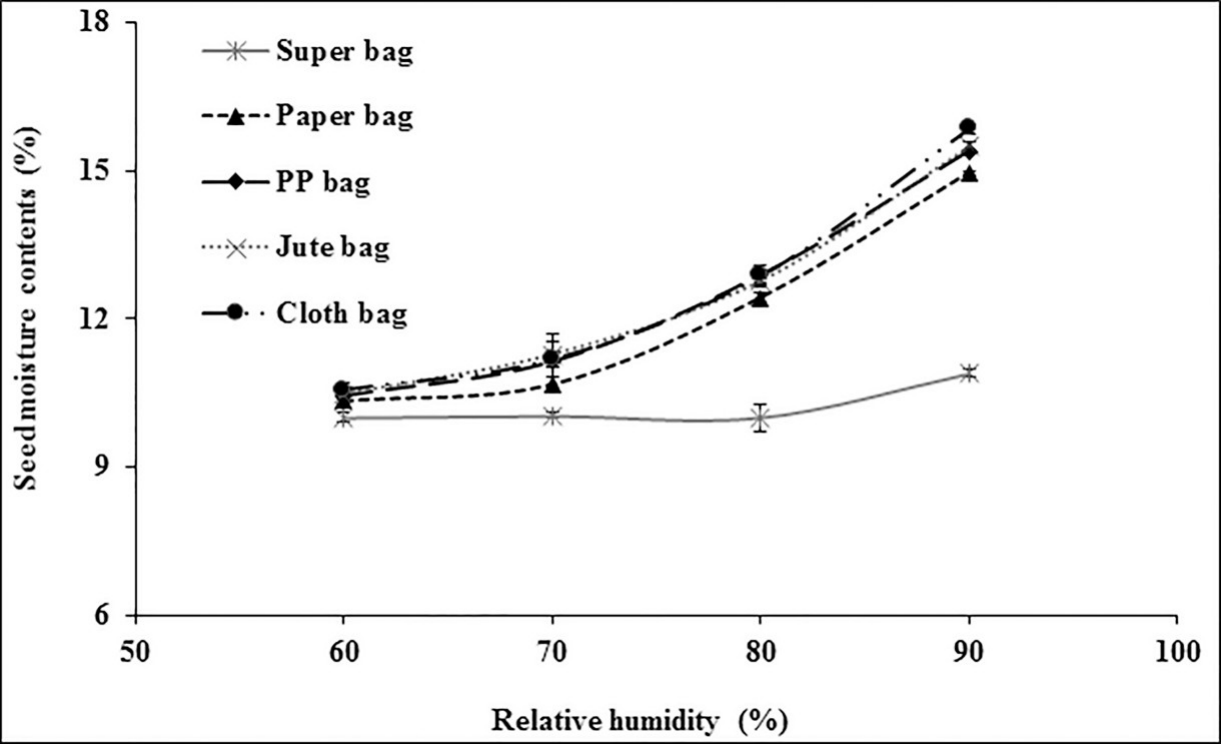
Seed moisture adsorption isotherms of maize seeds in different packaging materials at 25°C.

Within different packaging materials, maize seed had moisture contents in range of 9.99% (Super bag) to 10.56% (Cloth bag) at 60% RH. There was no significant difference in moisture contents of maize seed placed in conventional packaging materials at 60% RH (Fig 3). Similarly, moisture contents were lower in the seeds when placed in Super bag and paper bag at 70% RH while rest all bags have high seed moisture. At 80% RH, Super Bag maintained minimum seed moisture contents while seed in cloth and PP had maximum seed moisture contents. At 90% RH, although seed moisture contents increased from 10% (at 80% RH) to 10.89% in Super Bag but this increase was far less than that was observed for seed moisture in other packaging materials. Seed in cloth bag had maximum seed moisture contents (15.86%). Overall, moisture isotherms indicate that Super Bag maintained seed moisture at all levels of RH while seed moisture contents varied linearly with prevailing RH in all bags especially in cloth, PP and jute bag (Fig 3).

**Fig 4:**
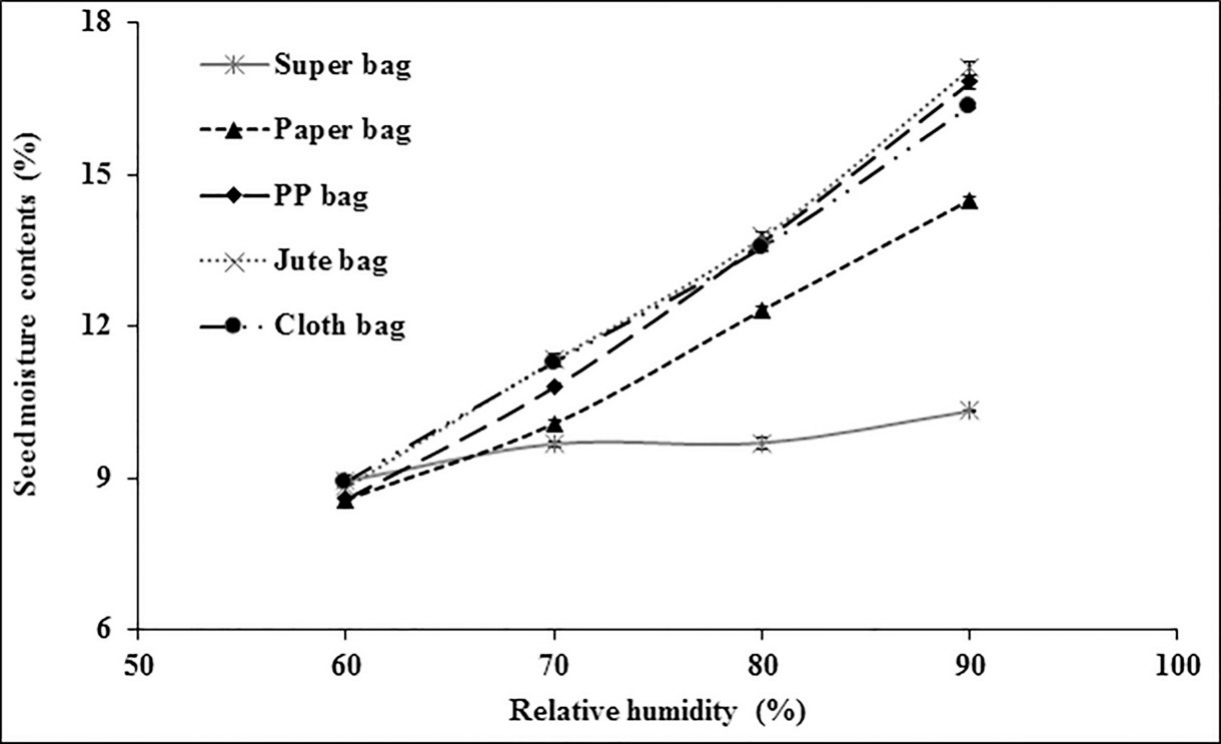
Seed moisture adsorption isotherms of wheat seeds in different packaging materials at 25°C.

There was no significant difference in moisture contents of wheat in all packaging materials at 60% RH (Fig 4). At 70% RH, Super Bag and paper bag had seeds with lowest moisture contents while seed in cloth and jute bag had higher moisture. Moisture contents of wheat seeds were strikingly different in various packaging materials at 80% RH. Super Bag had seed with minimum moisture contents followed by seeds moisture in paper bag. Rest all bags had higher seed moisture contents. At 90% RH, there was abrupt increase in moisture contents of wheat seed in Super Bag as well. Seed moisture contents increased from 9.69% (at 80% RH) to 10.32% in Super Bag. Seed in cloth bag had maximum seed moisture (17.1%).

**Fig 5:**
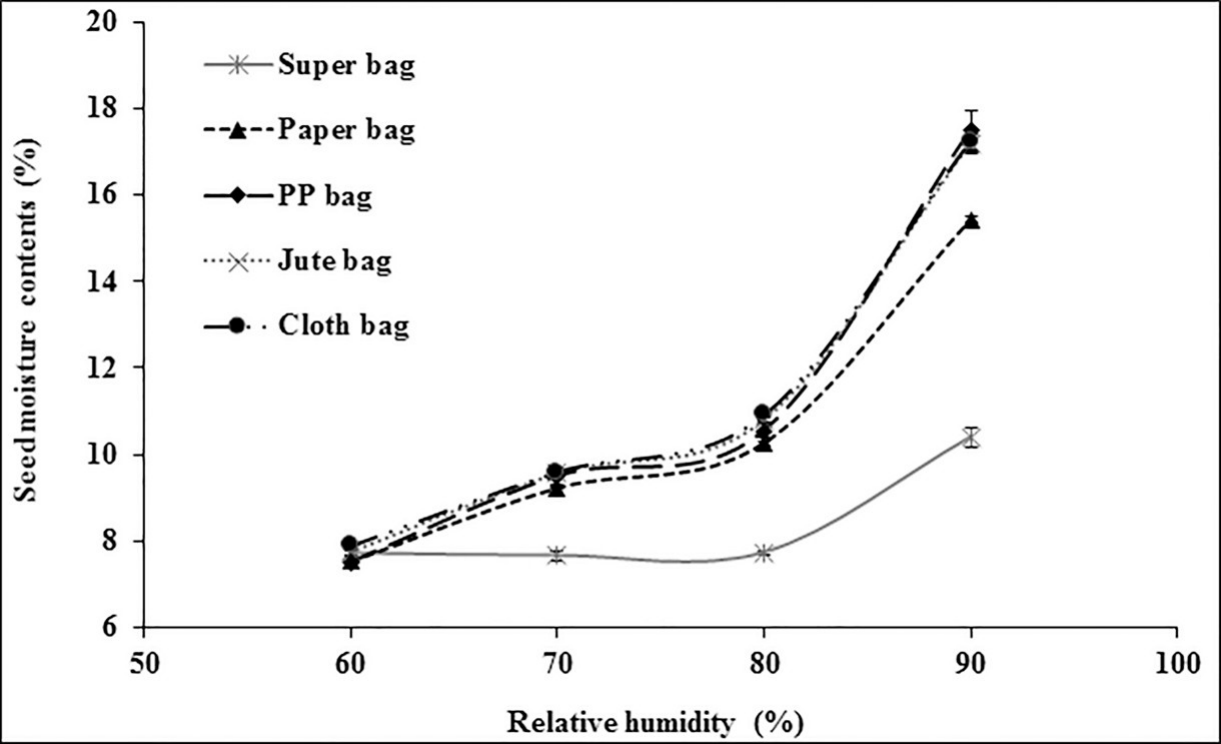
Seed moisture adsorption isotherms of cotton seeds in different packaging materials at 25°C.

At 60% RH, maximum cotton seed moisture was recorded in cloth bag while minimum seed moisture was observed in PP and paper bags. Seeds in Super Bag had minimum moisture contents compared the seeds in all other packaging materials at 70% RH (Fig 5). Moisture contents of cotton seeds at 80% RH were significantly higher in cloth bag while Super Bag had seeds with lowest moisture. Cotton seed moisture also increased in Super Bag at 90% RH but still considerably low (10.39%) as compared to seed moisture in rest of packaging materials (Fig 5). Seed in PP bag had maximum seed moisture (17.52%) that was statistically similar to cloth bag (17.26%) and jute bag (17.20%) followed by paper bag (15.43%).

**Fig 6:**
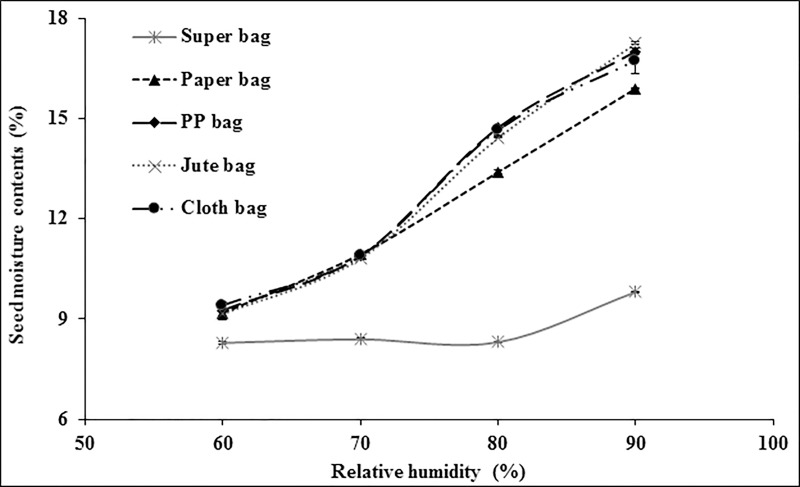
Seed moisture adsorption isotherms of quinoa seeds in different packaging materials at 25°C.

There was a considerable difference in moisture contents of quinoa seeds placed in different packaging material at 60% RH. Seeds in Super Bag had minimum moisture contents compared to the seeds in all other storage materials (Fig 6). At 70% RH, Super Bag had seeds with minimum moisture contents while seed in paper, jute, cloth and PP bag had high moisture. Moisture contents of quinoa seeds at 80% RH were in order of Super Bag < paper bag < jute bag < cloth bag < PP bag (Fig 6). At 90% RH, seed moisture contents increased from 8.30% (at 80% RH) to 9.81% in Super Bag but compared to other bags this increment was far less than that was noted in other bags. Seed in jute bag had maximum seed moisture (17.25%) followed by PP bag (17%) and cloth bag (16.73%).

### Seed storage study

Seed germination was highly linked with the initial seed moisture contents and storage conditions. Moreover, type of packaging materials also significantly affected seed germination of all crops under study. Highest germination (85%) of maize seed was recorded when it was stored in Super Bag at 8% initial seed moisture contents (SMC). Even in Super Bag, maize seed lost its germination completely when it was stored at 14% initial SMC. Likewise seed germination after 18 month of storage drastically reduced in all conventional packaging materials (Paper, PP, jute and cloth bag) irrespective of initial seed moisture contents (Table 2). Wheat seed germination was fairly good in all packaging materials with maximum (99%) germination in Super Bag after 18 month of storage at 8% initial seed moisture contents. Wheat seeds stored in all other conventional packaging materials at 8% initial SMC also had statistically similar germination percentage. Wheat seed stored in Super bag at 14% initial SMC had lowest (18%) germination, however, germination declined only slightly in conventional bags when stored at 14% initial SMC (Table 2).

**Table 2:** Final germination of crop seeds after 18 months of storage in different packaging materials at various levels of initial seed moisture contents.

Packaging Materials	Initial Germination (%)	Final Germination at 8% SMC	Final Germination at 14% SMC	Initial Germination (%)	Final Germination at 8% SMC	Final Germination at 14% SMC
	Maize			Wheat		
**Super Bag**	99	85±2.89a^z^	0±0 b	99.8	99±0.50 a	18±1.67 b
**Paper bag**	99	8±1.67 b	0±0 b	99.8	95±5.01 a	88±4.41 a
**PP bag**	99	3±1.67 b	2±2.89 b	99.8	88±2.89 a	82±1.67 a
**Jute Bag**	99	3±2.89 b	2±1.67 b	99.8	85±11.68 a	78±7.65 a
**Cloth Bag**	99	8±4.42 b	2±1.67 b	99.8	87±3.34 a	83±8.83 a
**HSD at P ≤ 0.05**	11			31		
	**Quinoa**			**Cotton**		
**Super Bag**	80.5	50±2.89 a	0±0 d	75	69±0.58 a	0±0 d
**Paper bag**	80.5	30±1.73 b	16±1.67 c	75	62±0.88ab	7±2.41 d
**PP bag**	80.5	23±4.41bc	17±1.53 c	75	58± 0.67b	28±3.47 c
**Jute Bag**	80.5	21±3.34bc	18±3.34 bc	75	59±1 b	36±1.16 b
**Cloth Bag**	80.5	23±1.67bc	20±2.89bc	75	60±0.66ab	29±3.53c
**HSD at P ≤ 0.05**	12			9		

Means within same column not sharing same letters are significantly different at p ≤ 0.05.

^z^Mean separation within columns by Tukey’s HSD at p ≤ 0.05. PP; Polypropylene, SMC; Seed moisture contents.

Cotton seed stored in Super Bag at 8% initial SMC gave maximum germination (68%) followed by germination (62%) of seed stored in paper bag after 18 months. Minimum seed germination was recorded when cotton seed was stored in Super Bag at 14% initial seed moisture contents. Seed storage in conventional porous packaging materials at 8% initial seed moisture contents, resulted into ≈ 15% losses in germination while these losses were higher (40–60%) when stored at 14% initial seed moisture contents (Table 2). Quinoa seed germination was drastically affected in all packaging materials at both 8 and 14% seed moisture contents. After 18 month of storage, highest germination (50%) was recorded for the seeds stored in Super Bag at 8% initial SMC. Seed storage in conventional packaging materials at 8% initial SMC, resulted into 50–60% losses in germination as compared to germination losses (30%) in Super Bag at 8% initial SMC. Quinoa seed completely lost its germination when stored in Super Bag at 14% initial SMC.

## Discussion

Due to its hygroscopic nature seed absorbs and desorbs moisture according to ambient relative humidity and attain equilibrium [9]. Seed moisture contents vary continuously according to RH and temperature of the environment. Extremely low water potential within the dry seeds creates greater affinity for the water molecule to readily absorb by the seeds. Seed moisture isotherms describe equilibrium relationship between the seed moisture contents and equilibrium RH at a given temperature [20]. If storage environment is a closed container, the seeds tend to equilibrate itself with that microclimate and RH of that closed container is known as equilibrium relative humidity. Seed moisture contents varied in different packaging materials due to varying water vapor transmission rates [12]. For all crop seeds, Super Bag had lower moisture contents at all levels of RH (Figs 3–6). This low moisture content is due to the hermetic nature of the Super Bag that created hindrance to the incoming water vapors even at higher RH. Super Bag is made up of multilayers of polyethylene having a less permeable barrier layer to prevent exchange of moisture and air and has very low vapor transmission rate i.e. <5 gm^-2^day^-1^ [14]. Seed moisture increased slightly in Super Bag at higher RH levels (80 and 90%).

Moisture contents in all conventional bags varied according to the ambient RH and continuously increased with increasing RH. Most of the crop seeds had highest moisture in cloth bag. Free exchange of moisture between seed and environment resulted into high moisture of the seeds at higher RH (Figs 3–6). Likewise, seed moisture contents were also higher when seeds were packed in jute and cloth bags. Large pore size of the jute, cloth and polypropylene bags provided free access to the water vapors which were readily absorbed by the seeds and ultimately elevated seed moisture contents.

Seed moisture isotherm also varies with seed oil contents [21]. Results of current study indicated that at a given level of RH there was a difference in seed moisture contents of different crops in same bag. For example, at 90% RH, moisture contents of contents of maize seeds were 15.86, 15.50 and 15.39% whereas wheat seed moisture contents were 16.35, 17.10, and 16.83 in cloth, jute and PP bag respectively (Figs 3 and 4). This difference in seed moisture contents for both crop seeds is due to difference in seed composition as wheat seed has ≈2.3% oil contents and 12% protein contents [22] whereas maize seed has 5% oil contents and 9% protein contents [23]. Wheat seed has more protein contents and protein has greater affinity for water molecule due to presence of positive and negative charges that provide site for hydrogen bonding [21] thus had higher moisture contents. Moreover, maize seeds have more oil contents as compared to wheat seeds and lipids have less affinity for the water molecule so low oil contents and higher protein contents were responsible for the elevated moisture contents in wheat seeds in this study (Figs 3 and 4).

Seed deterioration and aging is a continuous process and its ultimate effects are loss of seed viability and vigor. High seed moisture content is the major culprit that can speed up the process of seed deterioration. High germination in Super Bag at 8% initial SMC was the outcome of Dry Chain that was continuously maintained by the hermetic nature of Super Bag. Reduction of seed moisture by initial drying and packaging in moisture proof containers can reduce the deterioration of commodities by fungi, storage losses due to insects and thus increase the shelf life seeds [1, 6]. Seeds stored in Super Bag at 14% initial SMC rapidly lost their germination as they were unable to lose their moisture due to barrier layer of the Super Bag and those continuous high moisture contents proved lethal for the seed germination. Major reason for the reduction of seed germination in conventional packaging materials could be the high seed moisture contents that lead to the production of reactive oxygen species causing oxidative damage and seed deterioration [24]. Fluctuations in RH of the storage environment affect seed moisture contents as is evident from our seed moisture adsorption isotherm study. Seed absorb moisture under conditions of high ambient relative humidity and quickly lost its germination due to the deteriorative process occurring at faster rate [25].

## Conclusion

Hermetic Super Bag maintained seed moisture up to 70% RH. At higher level of RH, moisture contents increased to some extent (1–2%) in Super Bag, whereas this increase was much higher in conventional packaging materials (≈9% higher than original SMC at 90% RH). Seed storage in Super Bag at 8% SMC can help to prevent the significant increase in seed moisture at higher RH that will ultimately maintain high seed quality by implementing The Dry Chain. Seed storage in Super Bag at 14% SMC is not recommended as it resulted in significant germination losses due to rise in seed metabolic activities. Thus, it is highly recommended that seed must be dried to safe moisture limits before storage and should be maintained this dryness throughout supply chain.
